# *Diabrotica undecimpunctata virus 2*, a Novel Small RNA Virus Discovered from Southern Corn Rootworm, *Diabrotica undecimpunctata howardi* Barber (Coleoptera: Chrysomelidae)

**DOI:** 10.1128/MRA.00380-20

**Published:** 2020-06-25

**Authors:** Sijun Liu, Arnubio Valencia-Jiménez, Molly Darlington, Ana M. Vélez, Bryony C. Bonning

**Affiliations:** a Department of Entomology, Iowa State University, Ames, Iowa, USA; b Departamento de Producción Agropecuaria, Universidad de Caldas, Manizales, Colombia; c Department of Entomology, University of Nebraska—Lincoln, Lincoln, Nebraska, USA; d Department of Entomology and Nematology, University of Florida, Gainesville, Florida, USA; Portland State University

## Abstract

The genome of *Diabrotica undecimpunctata virus 2* (DuV2), a putative positive-sense, single-stranded RNA virus identified from the southern corn rootworm transcriptome, comprises 5,313 nucleotides, including a short poly(A) tail. The two open reading frames encode a nonstructural polyprotein (p156) and a putative capsid protein (p25).

## ANNOUNCEMENT

The southern corn rootworm (SCR), Diabrotica undecimpunctata howardi Barber, is one of three corn rootworm species that cause significant damage to agricultural and horticultural crops (1). Several small RNA viruses have been identified from the western corn rootworm, Diabrotica virgifera virgifera (2–4), but only one has been described from SCR to date (5). Here, we report on the genome of a second RNA virus identified from the transcriptome of SCR.

Eggs, larvae (first, second, and third instar), pupae, and adult (male and female) SCR were supplied by Crop Characteristics (Farmington, MN). The RNeasy minikit (Qiagen, Valencia, CA, USA) was used for isolation of total RNA from three biological replicates of 100 eggs, 50 first-instar larvae, 5 each of second- and third-instar larvae, 1 pupa, 1 adult female, and 1 adult male. cDNA libraries were constructed using the NEBNext Ultra RNA library prep kit for Illumina (NEB) for each stage-specific sample with poly(A) selection and paired-end sequencing with 150-nucleotide (nt) reads conducted on a HiSeq 2500 platform (Illumina, San Diego, CA). One or two replicate sequence data sets from each developmental stage were used for virus sequence discovery (5).

The Trinity assembler v2.6.6 (6) was used for assembly of high-quality reads. Default parameters were used for all software unless otherwise noted. A putative viral contig, with a short poly(A) tail, has 5,312 nt with a nucleotide composition of 37% G+C content. A total of 29,036 and 29,694 reads from two first-instar replicate data sets (comprising 50.7 million and 48.1 million reads and quality scores of 37.5 and 37.6, respectively) mapped to the viral contig with coverages of 819.8- and 838.3-fold, respectively. The virus is provisionally named *Diabrotica undecimpunctata virus 2* (DuV2). Sequences derived from DuV2 were observed in transcriptome data sets from all developmental stages of SCR. The near-full-length sequences were assembled from first-instar larvae, suggesting increased abundance and active replication of DuV2 in larvae relative to other developmental stages. The 5′ coding sequence located between nt 156 and 4286 encodes a putative nonstructural protein of 1,376 amino acids (aa) (156.1 kDa, p156), while the more 3′ sequence between nt 4366 and 5025 encodes a putative structural protein with 219 aa (25 kDa, p25). A conserved protein domain search revealed that p156 contains three conserved domains, two overlapping viral helicase domains (NCBI accession no. pfam01443, aa 598 to 826; cd17914, aa 593 to 726) (7), and one RNA-dependent RNA polymerase domain (RdRP_2; pfam00978). A *Tomato mosaic virus* coat protein domain (TMV_coat domain pfam00721) was identified in protein p25, supporting annotation as a capsid protein.

**FIG 1:**
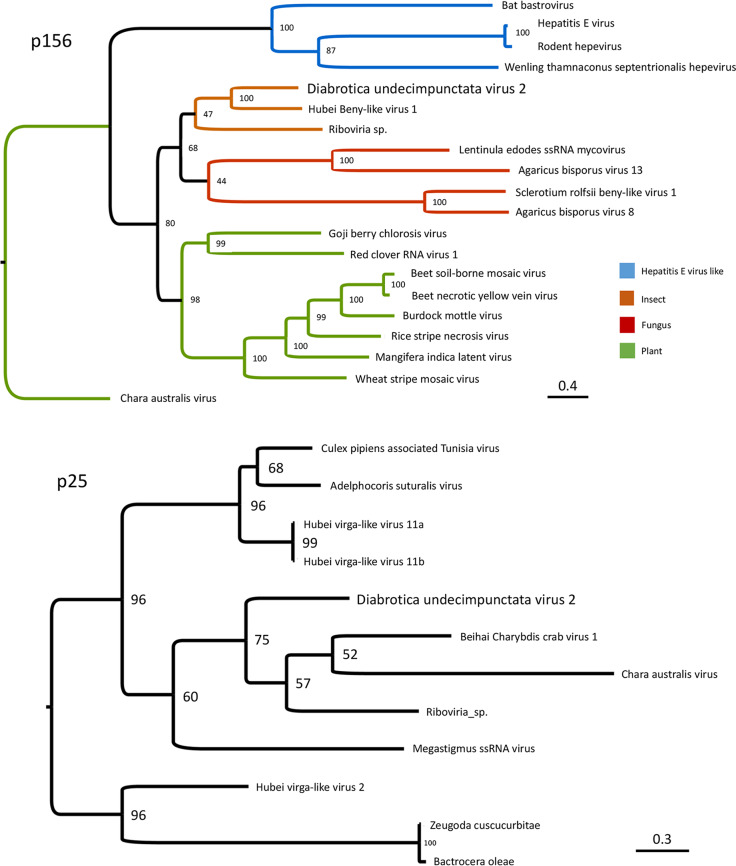
Phylogenetic analysis of DuV2 putative nonstructural protein p156 and putative structural protein p25. The replication-associated p156 is homologous to proteins from viruses with various hosts, including plants, fungi, insects, and hepatitis E-like viruses. Sequences with similarity to the capsid protein p25 were identified from viruses isolated from arthropods, although p25 also has a plant viral coat protein TMV domain. Viral proteins used for construction of the phylogenetic tree were selected from BLASTp analyses. Sequences were aligned with MAFFT v7.4.07 (using the AUTO strategy), and the maximum likelihood (ML) method was used for construction of the phylogenetic tree. ModelFinder implemented in IQ-TREE was used to identify the best partitioning scheme and models. ML analysis was performed using IQ-TREE with 10,000 ultrafast bootstrap replicates (10). Interactive Tree of Life (http://itol.embl.de) was used for visualization and annotation. The viruses and associated NCBI genome accession numbers are *Adelphocoris suturalis virus*, YP_009336480.1; *Agaricus bisporus virus 8*, AQM49930.1; *Agaricus bisporus virus 13*, AQM49941.1; *Bactrocera oleae*, XP_014085601.1; *Bat bastrovirus*, AWV67082.1; *Beet necrotic yellow vein virus*, ACA63016.1; *Beet soil-borne mosaic virus*, YP_009513207.1; *Beihai charybdis crab virus 1*, YP_009333243.1; *Burdock mottle virus*, YP_008219063.1; *Chara australis virus*, AEJ33768.1 and AEJ33771.1; Culex pipiens *associated Tunisia virus*, YP_009553258.1; *Goji berry chlorosis virus*, AYO99569.1; *Hepatitis E virus*, BAO47889.1; *Hubei beny-like virus 1*, APG77690.1; *Hubei virga-like virus 2*, YP_009337413.1; Hubei virga-like virus 11, AVK59472.1 and YP_009337245.1; Lentinula edodes *ssRNA mycovirus*, BAS04359.1; Mangifera indica *latent virus*, AMQ23297.1; *Megastigmus ssRNA virus*, QDZ71187.1; *Red clover RNA virus 1*, AVD98637.1; *Riboviria* sp. QDH86483.1; *Rice stripe necrosis virus*, QAR21011.1; *Rodent hepevirus*, AVW79978.1; *Sclerotium rolfsii beny-like virus 1*, AZF86092.1; *Wenling thamnaconus septentrionalis hepevirus*, AVM87557.1; *Wheat stripe mosaic virus*, AYD38100.1; and *Zeugodacus cucurbitae*; XP_011177481.1.

The highest BLASTp score for p156 (8) was *Hubei beny-like virus 1* (GenBank accession no. KX883789.1), an RNA virus isolated from dipteran insects (9) with 76% sequence coverage and 49% amino acid identity. Proteins similar to p156 included those from viruses of various host species (including fungi and plants) and soil samples (*Riboviria* sp.) (Fig. 1). BLASTp analysis for p25 identified the putative capsid protein of *Beihai charybdis crab virus 1* (GenBank accession no. YP_009333243.1), which has a genomic structure similar to that of DuV2.

### Data availability.

The genome sequence of DuV2 is available in GenBank under accession no. MN646771. The raw reads for SRA accession no. SRX8018653 to SRX8018656 are available at BioProject no. PRJNA615920.
